# Signal and morphological changes in the endolymph of patients with vestibular schwannoma on non-contrast 3D FLAIR at 3 Tesla

**DOI:** 10.1186/s12880-021-00670-9

**Published:** 2021-09-25

**Authors:** Iichiro Osawa, Eito Kozawa, Sayuri Tanaka, Akane Kaizu, Kaiji Inoue, Tetsuo Ikezono, Takamitsu Fujimaki, Mamoru Niitsu

**Affiliations:** 1 Department of Radiology, Saitama Medical University Hospital, 38 Morohongo, Moroyama-machi, Iruma-gun, Saitama, 350-0495 Japan; 2 Department of Otorhinolaryngology, Saitama Medical University Hospital, 38 Morohongo, Moroyama-machi, Iruma-gun, Saitama, 350-0495 Japan; 3 Department of Neurosurgery, Saitama Medical University Hospital, 38 Morohongo, Moroyama-machi, Iruma-gun, Saitama, 350-0495 Japan

**Keywords:** Vestibular schwannoma, Endolymphatic hydrops, 3D FLAIR, Magnetic resonance imaging (MRI), Vertigo

## Abstract

**Background:**

Non-contrast FLAIR revealed increased signal within the inner ear in patients with vestibular schwannoma, which is generally assumed to occur in the perilymph; however, the majority of previous studies did not differentiate between the endolymph and perilymph. Therefore, endolymph signal changes have not yet been investigated in detail. The purpose of the present study was three-fold: (1) to assess perilymph signal changes in patients with vestibular schwannoma on heavily T2-weighted (T2W) 3D FLAIR, also termed positive perilymphatic images (PPI), (2) to evaluate signal and morphological changes in the endolymph on PPI, and (3) to establish whether vertigo correlates with the signal intensity ratios (SIR) of the vestibular perilymph or vestibular endolymphatic hydrops.

**Methods:**

Forty-two patients with unilateral vestibular schwannoma were retrospectively recruited. We semi-quantitatively and qualitatively evaluated the perilymph signal intensity on the affected and unaffected sides. We also quantitatively examined the signal intensity of the vestibular perilymph and assessed the relationship between vertigo and the SIR of the vestibular perilymph on the affected side. We semi-quantitatively or qualitatively evaluated the endolymph, and investigated whether vestibular hydrops correlated with vertigo.

**Results:**

The perilymph on the affected side showed abnormal signal more frequently (signal intensity grade: overall mean 1.45 vs. 0.02; comparison of signal intensity: overall mean 36 vs. 0 cases) and in more parts (the entire inner ear vs. the basal turn of the cochlea and vestibule) than that on the unaffected side. No significant difference was observed in the SIR of the vestibular perilymph with and without vertigo (5.54 vs. 5.51, *p* = 0.18). The endolymph of the vestibule and semicircular canals showed the following characteristic features: no visualization (n = 4), signal change (n = 1), or vestibular hydrops (n = 10). A correlation was not observed between vestibular hydrops and vertigo (*p* = 1.000).

**Conclusions:**

PPI may provide useful information on signal and morphological changes in the endolymph of patients with vestibular schwannoma. Further research is warranted to clarify the relationship between vertigo and the MR features of the inner ear.

## Background

Vestibular schwannoma (also known as acoustic neuroma, acoustic neurilemoma, or acoustic neurinoma) is mostly a slow-growing benign tumor that may lead to cochlear and vestibular symptoms including hearing loss, tinnitus, ear fullness, and vertigo. Hearing loss is the most frequent symptom of vestibular schwannoma; however, 73% of patients with vestibular schwannoma develop tinnitus [1]. Vertigo, which is less common than hearing loss or tinnitus, severely affects the quality of life of patients with vestibular schwannoma [2] and is one of the predictors for its growth [3]. A previous study reported that 38% of patients with vestibular schwannoma presented with the full triad of vertigo, hearing loss, and tinnitus, mimicking Ménière's disease [4]. Although the exact mechanisms underlying these symptoms remain unclear, not only retrolabyrinthine dysfunction, but also inner ear dysfunction has been suggested to contribute to these symptoms [5, 6].

MR imaging is the technique of choice for the study of intracranial lesions [7–9], provides a unique insight into the microstructure of the inner ear and has revealed inner ear abnormalities in patients with vestibular schwannoma. Multiple attempts have been made to elucidate the relationship between the MR findings of the inner ear and audiovestibular dysfunctions in these patients [10–12]. Patients with unilateral vestibular schwannoma frequently showed increased signal intensity in the ipsilateral inner ear on 2D and 3D FLAIR images without contrast material [10–13]. In contrast, Silverstein et al. revealed increased protein levels in the cochlear perilymph of patients with vestibular schwannoma using a labyrinthine tap, which collected a sample of the perilymph through a hole in the stapes footplate [14]. These findings suggest that elevations in perilymph protein levels in the labyrinthine cause the T1 shortening of lymph fluid, resulting in high signal intensity on FLAIR images. However, the majority of MR studies mainly focused on the cochlea alone and did not provide sufficient information on the vestibule or semicircular canals. Moreover, the endolymph and perilymph were not differentiated and signal changes in the endolymph were not investigated in detail.

Naganawa et al. separately visualized the endolymph and perilymph in patients with vestibular schwannoma using non-contrast 3D FLAIR and identified cochlear and vestibular endolymphatic hydrops [15]. They also examined the relationship between vestibular endolymphatic hydrops and vertigo and found no correlation. Although this study focused on the vestibule and on morphological changes in the endolymph, it had a small sample size of 13 patients and did not utilize heavily T2-weighted (T2W) 3D FLAIR, as described below, which is more sensitive to increased signal intensity than 3D FLAIR. Naganawa et al. subsequently reported the utility of heavily T2W 3D FLAIR, also known as positive perilymphatic images (PPI), and heavily T2W 3D inversion recovery (T2W 3D IR), also termed positive endolymphatic images (PEI), to promote the recognition of the endolymphatic space in patients with Ménière's disease after the single-dose intravenous administration of gadolinium-based contrast material [16, 17]. Heavily T2W 3D FLAIR, which has been applied to the inner ear, brain, and spine [18–20], utilizes a heavily T2W sequence with a very long repetition time (TR). Lengthening of the TR enhances low-concentration gadolinium in fluid [21]; the T2 value of fluid containing low-concentration gadolinium is sufficiently long to be identified using the heavily T2W sequence [22]. Naganawa et al. previously reported that the contrast noise ratio (CNR) between the perilymph and endolymph was higher on heavily T2W 3D FLAIR than on conventional 3D FLAIR [22]. By subtracting PEI from PPI to generate HYDROPS (hybrid of reversed image of positive endolymph signal and native image of positive perilymph signal) images, it became possible to more easily detect the endolymphatic space [17]. Conventional 3D FLAIR cannot differentiate the endolymphatic space from surrounding bone and air, while HYDROPS distinguishes between the perilymph, endolymph, and bone in a single image. HYDROPS has been used with the intravenous administration of gadolinium to evaluate endolymphatic hydrops [17]. However, these novel techniques without the administration of gadolinium have not yet been applied to patients with vestibular schwannoma.

Therefore, the aim of the present study was three-fold: 1) to assess signal changes in the perilymph of patients with vestibular schwannoma on PPI, 2) to evaluate signal and morphological changes in the endolymph on PPI with the aid of PEI and HYDROPS, and 3) to establish whether vertigo correlates with the signal intensity ratio (SIR) of the vestibular perilymph or vestibular endolymphatic hydrops using a larger sample size.

## Methods

### Patients

This retrospective study was conducted at a single institution and approved by the Institutional Review Board of Saitama Medical University Hospital (18078.02). We obtained written informed consent for the procedures and opt-out consent for the use of retrospective clinical data from all patients. We consecutively enrolled 61 patients with unilateral vestibular schwannoma who underwent PPI and PEI using the 3 Tesla MR imaging unit (MAGNETOM Skyra, Siemens, Erlangen, Germany) with a 32-channel head coil (Siemens, Erlangen, Germany) between October 2016 and August 2018. Eighteen out of 61 patients underwent surgical treatment and/or stereotactic radiosurgery, 14 of whom received histopathological confirmation of a diagnosis of vestibular schwannoma. Nineteen patients were excluded because of the non-availability of imaging before surgery and radiosurgery (n = 17), cochlear schwannoma on the affected side (n = 1), and motion artifacts (n = 1). Therefore, 42 patients were retrospectively recruited (1 histopathologically and 41 radiologically diagnosed). We reviewed medical records to establish whether patients exhibited cochlear (hearing loss, tinnitus, and ear fullness) and vestibular (vertigo) symptoms. The clinical characteristics of the study population are summarized in Table 1.

**Table 1:** Characteristics of the study population (n = 42)

Male/female	16/26
Mean age (years)	60 (range 33–85, SD 13)
Hearing loss	26 (62%)
Tinnitus	29 (69%)
Ear fullness	11 (26%)
Vertigo	26 (62%)

*SD* standard deviation

### MR imaging

Forty-two patients underwent axial PPI, axial PEI, and axial T2W imaging of the inner ear. Axial PEI was obtained to generate HYDROPS images by subtracting PEI from PPI. All patients, except for one, underwent axial heavily T2W MR cisternography (MRC) for an anatomical reference of the total lymph fluid. PPI, PEI, and MRC were performed according to a protocol proposed by Naganawa et al. for the evaluation of endolymphatic hydrops [23]. Thirty-five out of 42 patients underwent sagittal or axial fat-suppressed T1-weighted (T1W) imaging immediately after the intravenous administration of gadolinium-based contrast material (Gadoteridol, ProHance, Eisai, Tokyo, Japan) in a single dose of 0.2 mL/kg (0.1 mmol/kg). Thirteen out of 42 patients underwent follow-up MR imaging at a mean of 7.4 months (range 1–12 months) after the baseline MR scan during the study period. MR imaging parameters are summarized in Table 2.

**Table 2:** MR imaging parameters

	Heavily T2W 3D FLAIR (PPI)	Heavily T2W 3D IR (PEI)	MR cisternography (MRC)	T2W imaging
TR/TE (ms)	9000/542	9000/542	4400/542	1300/123
Inversion time (ms)	2250	2050	−	−
Frequency-selective fat suppression prepulse	+	+	+	−
Flip angle	Average flip angle 120° followed by 90° restore pulse	Average flip angle 120° followed by 90° restore pulse	Average flip angle 120° followed by 90° restore pulse	Average flip angle 120° followed by 90° restore pulse
Echo train length	519	519	519	87
Acquisition matrix size	324 × 384	324 × 384	324 × 384	320 × 320
FOV (mm^2^)	166 × 196	166 × 196	166 × 196	200 × 200
Axial slices	104	104	104	160
In-plane resolution (mm)	0.5 × 0.5	0.5 × 0.5	0.5 × 0.5	0.3 × 0.3 (after zero-fill interpolation)
Slice thickness (mm)	1.0	1.0	1.0	0.3
Bandwidth (Hz/pixel)	434	434	434	446
Acceleration factor	2 using GRAPPA (in-plane right-left phase-encoding direction)	2 using GRAPPA (in-plane right-left phase-encoding direction)	2 using GRAPPA (in-plane right-left phase-encoding direction)	2 using GRAPPA (in-plane right-left phase-encoding direction)
Number of excitations	2	2	1.8	2
Phase partial Fourier acquisition	+	+	+	+
Slice partial Fourier acquisition	−	−	−	+
Total time (min.sec)	7.17	7.17	3.15	6.27

### Imaging analysis

Table 3 shows an overview of the imaging analysis. Images of the perilymph and endolymph were analyzed qualitatively, semi-quantitatively, or quantitatively by two board-certified radiologists with 9 and 29 years of experience who were blinded to clinical information. All images were independently reviewed by the two radiologists, and discrepancies in qualitative and semi-quantitative analyses were resolved through discussion to reach the final consensus, while quantitative data obtained by the two radiologists were presented as means and standard deviations (SD).

**Table 3:** Overview of imaging analyses

	Category	Side	Portion	Qualitative analysis	Semi-quantitative analysis	Quantitative analysis
Perilymph	Signal intensity	Affected and unaffected sides	Cochlea, vestibule, and semicircular canals	Comparison between affected and unaffected sides	Grading (3-point-scale)	–
		Affected sides	Vestibule	–	–	Signal intensity ratio (SIR)
Endolymph	Visualization	Affected side	Cochlea and vestibule	Visualization or no visualization	–	–
	Signal change	Affected side	Cochlea, vestibule, and semicircular canals	Presence of increased signal intensity	–	–
	Endolymphatic hydrops	Affected side	Cochlea and vestibule	–	Grading (3-point-scale)	–

#### Qualitative or semi-quantitative analysis

**Fig. 1:**
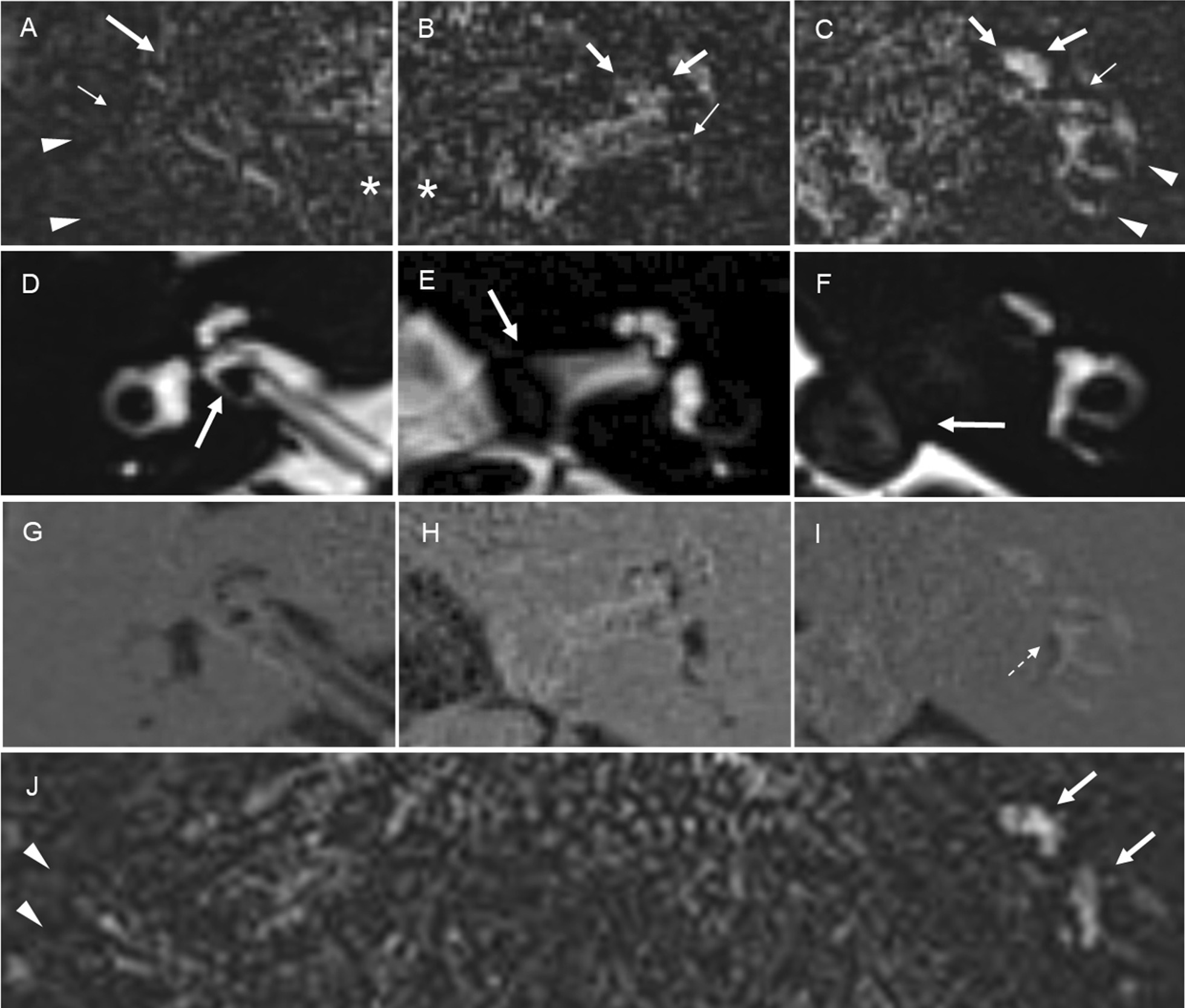
Examples of grading and comparisons of signal intensity of the perilymph: The first row represents PPI for grades 0 (**a**), 1 (**b**), and 2 (**c**). The second and third rows are the corresponding MRC (**d**–**f**) and HYDROPS (**g**–**i**) images, respectively. **a** PPI for grade 0: The signal intensity of the perilymph, including the entire cochlea (arrow), vestibule (small arrow), and lateral as well as posterior semicircular canals (arrowheads), was similar to that of CSF in the cerebellopontine angle cistern (asterisk). **b** PPI for grade 1: The signal intensity of the perilymph, including the cochlear basal and middle/apical turns (arrows), and vestibule (small arrow), was higher than that of CSF (asterisk) without sharply delineated borders. **c** PPI for grade 2: The signal intensity of the perilymph, including the entire cochlea (arrows), vestibule (small arrow), and lateral as well as posterior semicircular canals (arrowheads), was markedly higher than that of CSF with sharply delineated borders. The higher the grade on PPI, the higher the signal intensity of the perilymph was on corresponding HYDROPS images. **d**–**f** The corresponding MRC: Each arrow showed a vestibular schwannoma. **g**–**i** The corresponding HYDROPS: Of note, HYDROPS (**i**) more easily detected the vestibular endolymphatic space as hypointensity (dotted arrow) than the corresponding PPI for grade 2 (**c**). **j** Comparison of perilymph signal intensity between the affected and unaffected sides on PPI: The signal of the perilymph on the affected side (arrows) was higher than that on the unaffected side (arrowheads)

We qualitatively and semi-quantitatively evaluated the signal intensity of the perilymph on the affected and unaffected sides based on PPI. Figure 1 shows examples of grading and comparisons of the signal intensity of the perilymph. The perilymph was subdivided into the following six parts: the cochlear basal turn, cochlear middle/apical turn, vestibule, anterior semicircular canal, lateral semicircular canal, and posterior semicircular canal. The signal intensity of each perilymph on the affected and unaffected sides was graded using the following 3-point-scale: 0 = similar to that of cerebrospinal fluid (CSF) in the cerebellopontine angle cistern (normal signal), 1 = higher than that of CSF without sharply delineated borders, and 2 = markedly higher than that of CSF with sharply delineated borders. Grades 1 and 2 were defined as abnormal increased signal. In our experience, abnormal increased signal in the semicircular canals was hypothesized to reflect the perilymphatic signal because this signal was generally continuous with increased signal intensity at the level of the vestibular perilymph.

We qualitatively compared the signal intensity of the perilymph between the affected and unaffected sides on PPI and classified it into three groups: equal signal, higher signal on the affected side, and higher signal on the unaffected side. If the perilymph was not separately visualized from the endolymph, the signal intensity of entire lymphatic space was compared.

**Fig. 2:**
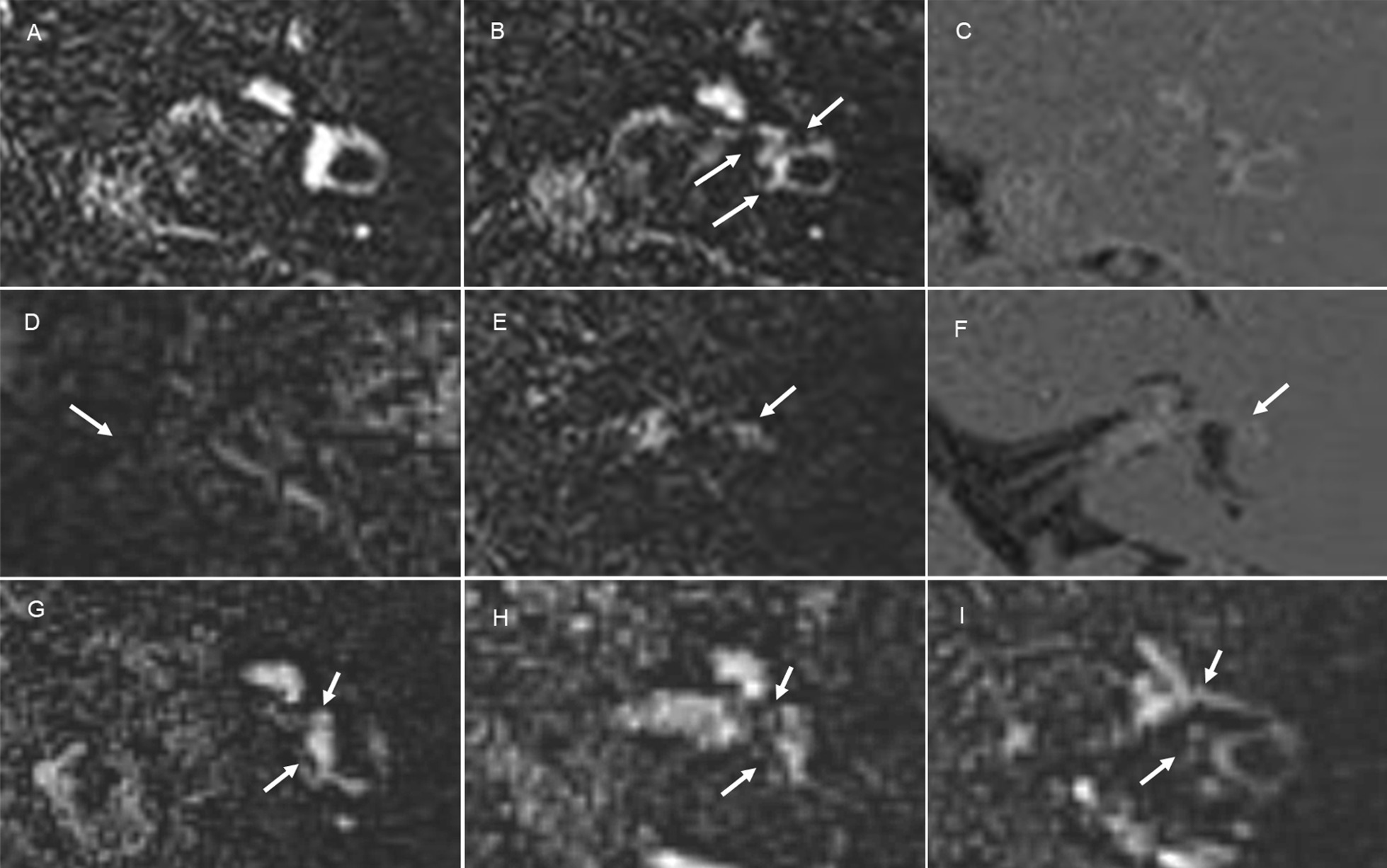
Examples of qualitative and semi-quantitative analyses of the endolymph on the affected side. **a**–**c** Visualization of the endolymph: The endolymphatic spaces of the vestibule and semicircular canals were not visualized on PPI for grade 2 (**a**), but were visualized on PPI for grade 2 (**b**, arrows) and the corresponding HYDROPS (**c**). **d**–**f** Signal change in the endolymph: The endolymph of the lateral semicircular canal showed no signal change on PPI for grade 0 (**d** same as Fig. 1a, arrow), whereas signal change was observed on PPI for grade 0 (**e**, arrow) as well as the corresponding HYDROPS (**f**, arrow). **g**–**i** Endolymphatic hydrops on PPI for grade 2: Vestibular hydrops was graded as no (**g**, arrows), mild (**h**, arrows), and significant (**i**, arrows)

The endolymph on the affected side was evaluated qualitatively or semi-quantitatively by focusing on the following three aspects: visualization, signal change, and endolymphatic hydrops. Figure 2 shows examples of qualitative (visualization and signal change) and semi-quantitative (endolymphatic hydrops) analyses. PPI was used to evaluate the endolymph with the aid of PEI and HYDROPS. The endolymph was classified into seven parts, including the cochlear duct of the basal turn, the cochlear duct of the middle/apical turn, the vestibule saccule, vestibule utricle, ampulla of the anterior semicircular canal, ampulla of the lateral semicircular canal, and ampulla of the posterior semicircular canal. Visualization of the endolymph on the affected side was qualitatively evaluated mainly in the cochlea and vestibule, and divided into two categories: visualized or not visualized. Visualization of the cochlear and vestibular endolymph was assessed when the signal intensity of the corresponding perilymph was interpreted as grade 2 on PPI because grades 0 and 1 on PPI did not show sufficiently increased signal intensity in the perilymph to identify the low signal of the endolymph.

The signal change in the endolymph was qualitatively assessed when the signal intensity of the corresponding perilymph was normal on PPI (grade 0). Increased signal intensity in the endolymph was interpreted as the presence of signal change.

We semi-quantitatively assessed endolymphatic hydrops of the cochlea and vestibule according to the criteria proposed by Nakashima et al. [24]. The grading system of cochlear endolymphatic hydrops was as follows: no = no bulging of the cochlear duct or no visualization of the cochlear duct, mild = the cochlear duct was bulging toward the scala vestibuli without exceeding the perilymphatic space of the scala vestibuli, significant = the cochlear duct exceeded the perilymphatic space of the scala vestibuli. Endolymphatic hydrops of the vestibule was graded as follows: no = less than 33% of the vestibule was occupied by the endolymph, mild = 33 to 50% of the vestibule was occupied by the endolymph, significant = more than 50% of the vestibule was occupied by the endolymph. Endolymphatic hydrops was assessed when the endolymph was visualized based on PPI. However, cochlear hydrops was graded as “no” when the endolymph was not visualized. Conversely, vestibular hydrops was not evaluated when neither the saccule nor utricle was visualized, while vestibular hydrops was graded when either the saccule or utricle was visualized. We also examined the relationship between vestibular endolymphatic hydrops and vertigo.

#### Quantitative analysis

**Fig. 3:**
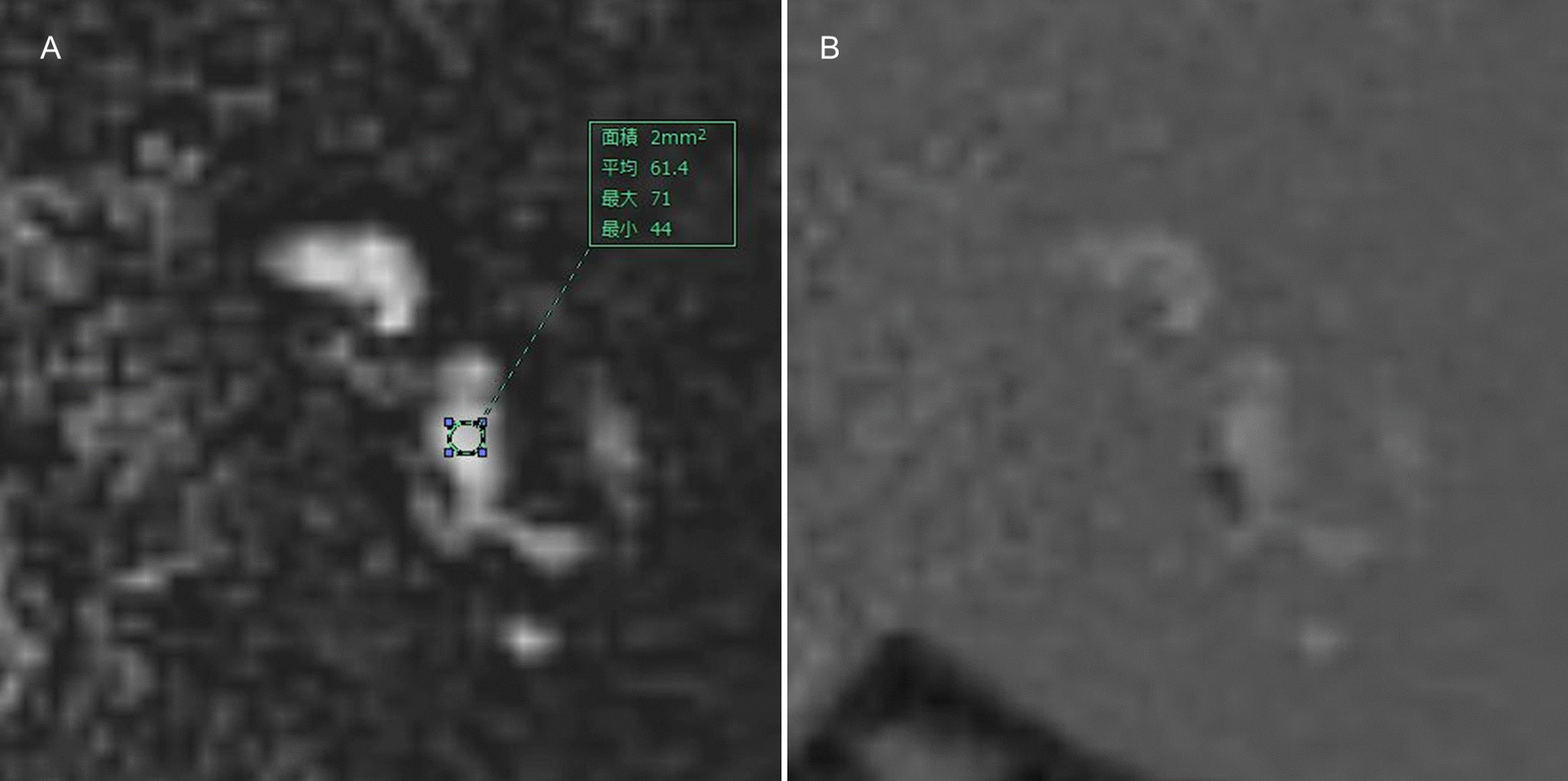
An example of the quantitative analysis of the vestibular perilymph. Regarding grade 2 plus visualization of the vestibular endolymph (**a**), a circular ROI was placed on the vestibular perilymph with reference to HYDROPS (**b**)

We quantitatively evaluated the signal intensity of the vestibular perilymph on the affected side based on heavily T2W 3D FLAIR using the workstation Synapse VINCENT version 5.2 (Fuji Film, Tokyo, Japan). Figure 3 shows an example of the quantitative analysis of the vestibular perilymph. The perilymph was classified into six parts and the endolymph was divided into seven parts as described above. When the signal intensity of the perilymph was interpreted as grade 2 and the endolymph was visualized on the affected side, it was possible to differentiate between the perilymph and endolymph. Under these conditions, a circular region of interest (ROI) (2 mm^2^) was selectively placed in the vestibular perilymph with reference to HYDROPS. A circular ROI (50 mm^2^) was then placed in the brain stem at the level of the internal auditory canal. We calculated the SIR using the following formula: SIR = signal intensity of the vestibule/signal intensity of the brain stem. We then assessed the relationship between vertigo and the SIR of the vestibular perilymph on PPI.

### Statistical analysis

The Wilcoxon signed-rank test was used to compare grading scales for signal intensity between the affected and unaffected sides. Welch’s *t*-test was employed to compare SIR on the affected side with and without vertigo. Fisher’s exact test was used to elucidate the relationship between vertigo and vestibular endolymphatic hydrops. Gwet’s AC1 and the intraclass correlation coefficient (ICC) (2, 1) were used as indices of interreader agreement. Gwet’s AC1 was used instead of assessing Cohen’s kappa because this method overcomes the limitation of kappa being sensitive to trait prevalence and marginal probability [25]. ICC (2, 1) was estimated with a two-way random effects model of absolute agreement for a single observation. AC1 and ICC values were interpreted according to the following classifications by Landis and Koch [26]: < 0, indicating no agreement; 0–0.20, slight agreement; 0.21–0.40, fair agreement; 0.41–0.60, moderate agreement; 0.61–0.80, substantial agreement; and 0.81–1, almost perfect agreement. All statistical calculations were conducted with SPSS 27.0 software (IBM, Armonk, NY, USA) and the statistical computing language R (Version 4.0.5; http//www.r-project.org/). *p* values less than 0.05 were considered to be significant.

## Results

### Perilymph

**Fig. 4:**
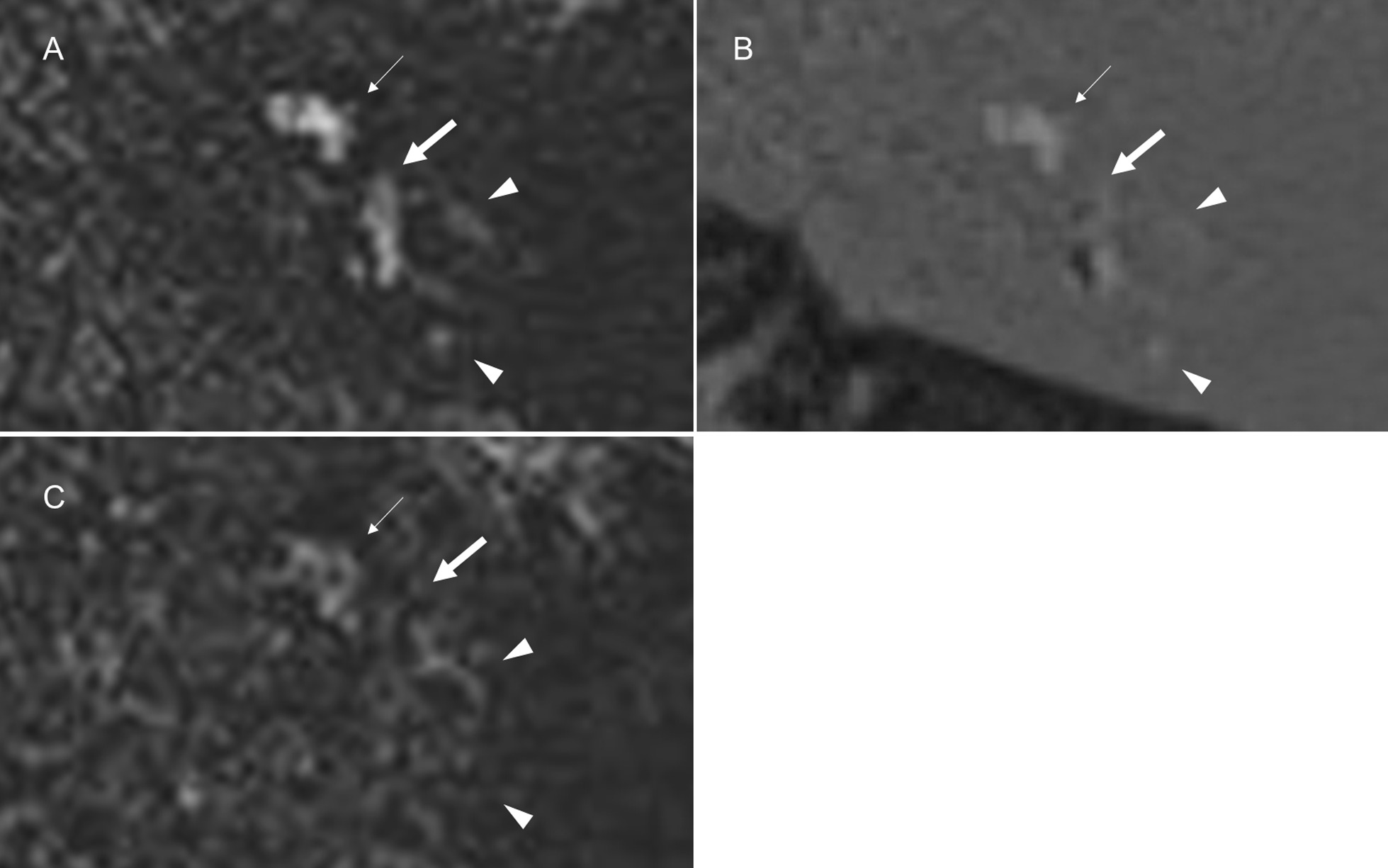
A 72-year-old female with left vestibular schwannoma showing serial changes in the perilymph. PPI (**a**) and HYDROPS (**b**) display increased signal intensity in the perilymph of the cochlea (small arrow), vestibule (arrow), and semicircular canals (arrowheads) on the affected side. The signal intensity in the perilymph decreased after one year on PPI (**c**)

Table 4 shows a summary of results for grading and comparisons of signal intensity between the affected and unaffected sides. Each perilymph on the affected side more frequently showed abnormal signal based on PPI and in more parts than that on the unaffected side. Out of 13 patients who underwent follow-up imaging, one patient, who had high signal intensity in the perilymph through the entire inner ear on the affected side based on PPI, exhibited decreased signal after one year (Fig. 4). Interreader agreement was substantial to almost perfect for the grading of signal intensity (AC1 = 0.75–1.00), and almost perfect for comparisons of signal intensity (AC1 = 0.94–0.99). Table 5 shows a summary of the results for the SIR of the vestibular perilymph on PPI. We enrolled 32 out of 42 patients to assess the SIR of the vestibular perilymph, after excluding 10 patients in whom it was not possible to distinguish between the perilymph and endolymph on the affected side under the following two conditions: 1) when the signal intensity of the perilymph was interpreted as grade 0 or 1 (n = 9), and 2) when the signal intensity of the perilymph was interpreted as grade 2 and the entire vestibular endolymph not visualized (n = 1). Interreader agreement was moderate for the SIR (ICC (2, 1) = 0.67, 95% confidence interval (CI) = 0.43–0.82). Table 6 shows comparisons of the SIR of the vestibular perilymph on the affected side with and without vertigo. No significant differences were observed in the SIR of the vestibular perilymph with and without vertigo on PPI (Welch’s *t*-test).

**Table 4:** Grading and comparisons of the signal intensity of the perilymph (n = 42)

	PPI (n = 42)								
	Grading of signal intensity					Comparisons of signal intensity between affected and unaffected sides			
Perilymph	Affected side Mean (SD)	Unaffected side Mean (SD)	AC1 value (95% CI) Affected side	AC1 value (95% CI) Unaffected side	*p* value (Wilcoxon signed-rank test)	Higher signal on the affected side	Equal signal	Higher signal on the unaffected side	AC1 value (95% CI)
Basal turn of CD	1.69 (0.64)	0.07 (0.26)	0.92 (0.83–1.02)	0.98 (0.96–1.00)	< .0001	38	4	0	0.97 (0.84–1.00)
Middle/apical turn of CD	1.67 (0.69)	0	0.93 (0.85–1.02)	1.00 (1.00–1.00)	< .0001	37	5	0	0.97 (0.93–1.00)
Vestibule	1.67 (0.69)	0.02 (0.15)	0.98 (0.95–1.01)	0.99 (0.98–1.01)	< .0001	37	4	1	0.94 (0.87–1.01)
Anterior SCC	1.41 (0.81)	0	0.75 (0.60–0.90)	1.00 (1.00–1.00)	< .0001	34	8	0	0.98 (0.96–1.01)
Lateral SCC	1.36 (0.79)	0	0.75 (0.60–0.90)	1.00 (1.00–1.00)	< .0001	33	9	0	0.99 (0.97–1.01)
Posterior SCC	1.45 (0.77)	0	0.86 (0.77–0.95)	0.99 (0.98–1.01)	< .0001	35	7	0	0.99 (0.97–1.01)
Overall mean	1.45	0.02				36	6	0	

*PPI* positive perilymphatic images, *SD* standard deviation, *CI* confidence interval, *CD* cochlear duct, *SCC* semicircular canal

Signal intensity was graded as follows: 0 = similar to that of CSF in the cerebellopontine angle cistern (normal signal), 1 = higher than that of CSF without sharply delineated borders, and 2 = markedly higher than that of CSF with sharply delineated borders

**Table 5:** SIR of the vestibular perilymph on PPI (n = 32)

	PPI (n = 32)	
	Affected side mean (SD)	ICC value (95% CI)
SIR of the vestibular perilymph	5.47 (1.38)	0.67 (0.43–0.82)

*SIR* signal intensity ratio, *PPI* positive perilymphatic images, *SD* standard deviation, *ICC* intraclass correlation coefficient, *CI* confidence interval

**Table 6:** Comparisons of SIR of the vestibular perilymph on the affected side with and without vertigo

	Vertigo mean (SD)		*p* value (Welch’s *t*-test)
	Yes (n = 18)	No (n = 14)	
SIR of the vestibular perilymph on PPI	5.45 (1.58)	5.51 (1.09)	0.18

*SIR* signal intensity ratio, *SD* standard deviation, *PPI* positive perilymphatic images

### Endolymph

**Fig. 5:**
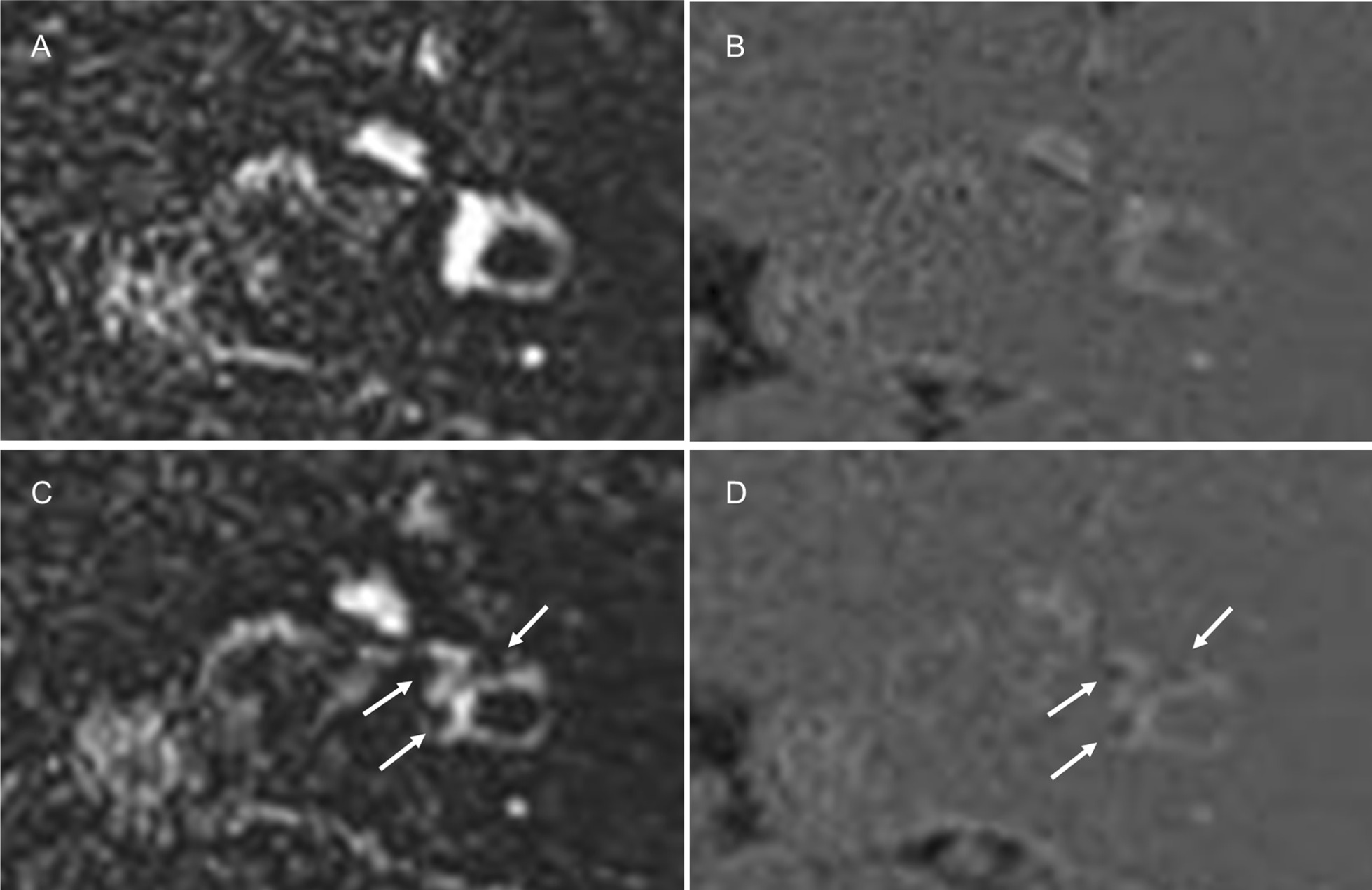
A 45-year-old male with left vestibular schwannoma. PPI (**a**) and HYDROPS (**b**) showed no visualization of the endolymph in the vestibule and semicircular canals on the affected side. The endolymph was visible after one year (**c** and **d**, arrows)

**Fig. 6:**
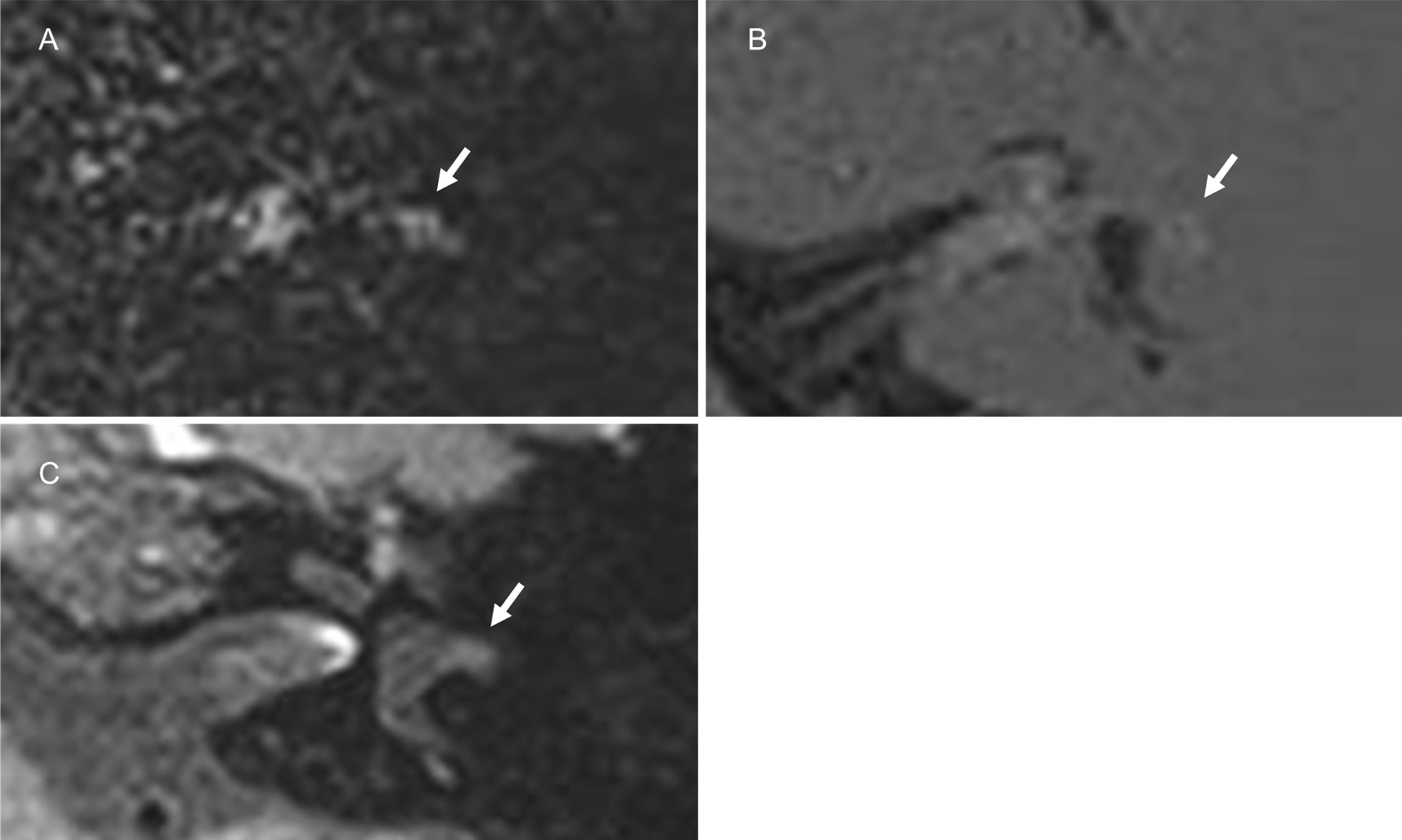
A 47-year-old female with left vestibular schwannoma. The ampulla of the lateral semicircular canal had high signal intensity on PPI (**a**, arrow) and HYDROPS (**b**, arrow), allowing for its easy recognition. A post-contrast T1W image showed faintly increased signal intensity in the ampulla (**c**, arrow)

Regarding visualization of the endolymph, we enrolled 33 out of 42 patients to evaluate the cochlear and vestibular endolymph on the affected side, after excluding the same nine patients who did not show sufficient signal intensity in the corresponding perilymph (grade 0 or 1 on PPI). In the cochlea, the endolymph was not identified on PPI. Interreader agreement was almost perfect for the visualization of the cochlear endolymph (AC1 = 1.00, 95% CI = 1.00–1.00). In contrast, the endolymph of the vestibule and semicircular canals showed the following abnormal findings: no visualization of the endolymph (n = 4) and signal change in the endolymph (n = 1). Among 11 of 33 patients, who underwent follow-up imaging, one patient showed no visualization of any parts of endolymph in the vestibule and semicircular canals. However, all parts of the endolymph were clearly identified after one year (Fig. 5). The three remaining patients showed no visualization of the endolymph in the saccule. Interreader agreement was almost perfect for the visualization of the vestibular endolymph (AC1 = 1.00, 95% CI = 1.00–1.00). Only one out of 42 patients, who presented with vertigo, showed abnormal signal intensity in the ampulla of the lateral and anterior semicircular canals, which depicted high intensity on PPI and low intensity on PEI without contrast enhancement (Fig. 6). A post-contrast T1W image showed faintly increased signal intensity in the ampulla; however, it remained unclear whether contrast enhancement occurred because pre-contrast T1W images were not obtained. Interreader agreement was almost perfect for the signal change in the endolymph (AC1 = 1.00, 95% CI = 1.00–1.00).

**Fig. 7:**
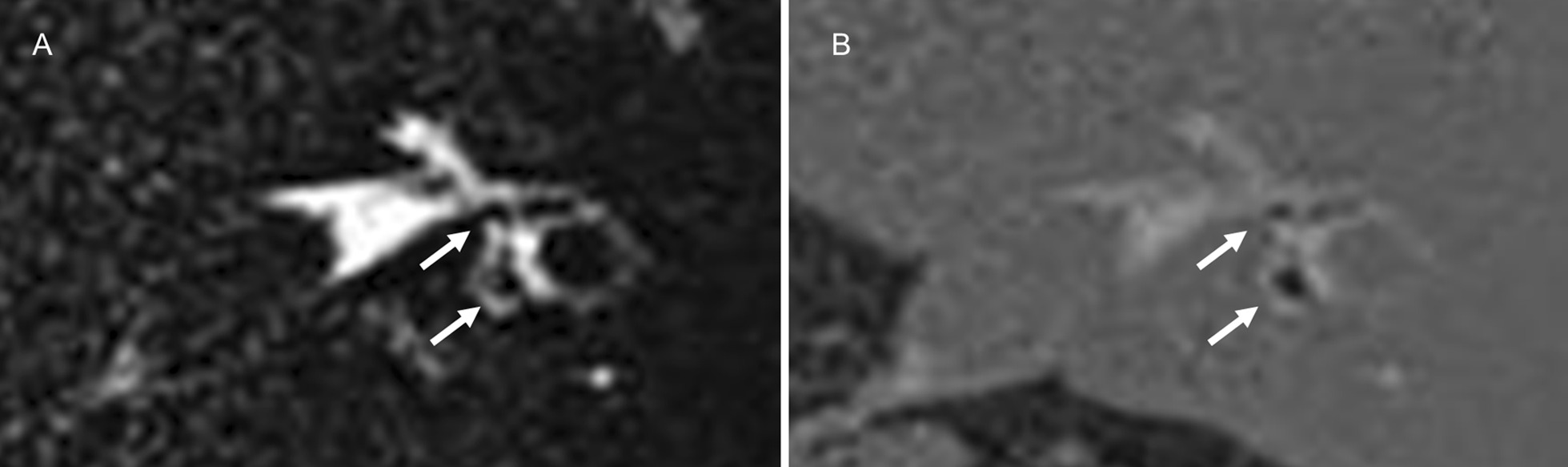
A 66-year-old male with left vestibular schwannoma showing vestibular endolymphatic hydrops. PPI (**a**) and HYDROPS (**b**) demonstrated mild hydrops in the vestibule (arrows)

Regarding endolymphatic hydrops, nine out of 42 patients were excluded from the evaluation of cochlear endolymphatic hydrops because the corresponding cochlear perilymph was interpreted as grade 0 or 1 on PPI. Cochlear endolymphatic hydrops was graded as “no” on the affected side in all of the 33 remaining patients because no endolymph was detected in the cochlea. Interreader agreement was almost perfect for the visualization of cochlear endolymphatic hydrops (AC1 = 1.00, 95% CI = 1.00–1.00). On the other hand, 10 out of 42 patients were excluded from the evaluation of vestibular endolymphatic hydrops due to grade 0 or 1 for the corresponding perilymph on PPI (n = 9) and no visualization of the entire vestibular endolymph (n = 1). Among the 32 remaining patients, vestibular hydrops was graded as “no” in 22, “mild” in 8, and “significant” in 2 (Fig. 7). Interreader agreement was substantial for vestibular endolymphatic hydrops (AC1 = 0.72, 95% CI = 0.50–0.91). A correlation was not observed between vestibular endolymphatic hydrops and vertigo (Fisher’s exact test, *p* = 1.000).

## Discussion

We herein revealed that the perilymph on the affected side more frequently showed abnormal signal and in more parts than that on the unaffected side in patients with vestibular schwannoma. However, no significant difference was observed in the SIR of the vestibular perilymph with and without vertigo. We also demonstrated signal and morphological changes in the vestibular and semicircular canal endolymph of patients with vestibular schwannoma. To the best of our knowledge, this is the first study to describe chronological alterations in the perilymph and endolymph. We separated the perilymphatic and endolymphatic spaces in patients with vestibular schwannoma using 3D FLAIR and 3D IR-based images. As a result, we were able to evaluate the signal intensity of the perilymph and endolymph separately and elucidate the endolymphatic morphology.

The present results were consistent with previous findings reported by Lee et al., which showed increased signal intensity in more than 90% of the vestibule and cochlea on the affected side using the 3D FLAIR sequence [10]. The present study demonstrated a higher prevalence of signal alterations in semicircular canals on the affected side than that reported previously [13], which may be due to the higher resolution and sensitivity to signal changes on heavily T2W 3D FLAIR.

It is crucial to separately visualize the perilymphatic and endolymphatic spaces for the accurate assessment of the inner ear. If ROI is placed without discriminating between the endolymph and perilymph to evaluate the signal intensity of the perilymph, the signal or morphology of the endolymph may affect signal intensity measurements.

Characteristic features observed in the endolymph of the vestibule and semicircular canals include the following: (1) no visualization of the endolymph, (2) signal change in the endolymph, and (3) endolymphatic hydrops. In one case, the endolymph was not identified in the vestibule as well as in the three semicircular canals, but was clearly visible on follow-up MR images. Three hypotheses have been proposed to explain why the endolymph was not visualized: (1) endolymph collapse, (2) abnormal signal in the endolymph with intralabyrinthine fistula or rupture, and (3) abnormal signal in the endolymph without intralabyrinthine fistula or rupture. This result suggests that lymphatic conditions in the inner ear of patients with vestibular schwannoma markedly change. If this change correlates with inner ear function, it may be important to evaluate serial changes in the endolymphatic signal as an indicator of function. Furthermore, only one patient, who presented with vertigo, showed signal alteration in the ampulla of the lateral and anterior semicircular canals based on PPI and PEI. Naganawa et al. reported that a patient receiving anticoagulant therapy developed vertigo and underwent 3D FLAIR imaging, which demonstrated increased signal intensity in the ampulla of the lateral and posterior semicircular canals, presumably due to hemorrhage [27]. Another study reported signal change in the vestibular endolymph in patients with vestibular schwannoma using fast imaging employing steady state acquisition with cycle phase (FIESTA-C) without gadolinium administration [28]. Although the pathology of T1-shortening observed in our case was unclear, this signal alteration may correlate with vertigo. The present study also revealed vestibular endolymphatic hydrops in ten out of 32 patients who were enrolled to assess vestibular hydrops. In contrast to the study conducted by Naganawa et al. [15], the present study examined a larger number of patients with vestibular schwannoma using PPI with better sensitivity and HYDROPS with better CNR. Although we also excluded patients without sufficiently increased signal intensity in the perilymph to identify the low signal of the endolymph using the grading systems, the results obtained were consistent with previous findings.

Cochlear and vestibular symptoms were generally considered to occur as a result of retrolabyrinthine dysfunction caused by tumor compression of the vestibulocochlear nerve. However, recent studies provided evidence to suggest that the origin of these symptoms may be partially explained by inner ear dysfunction [5, 6]. For example, a histopathological study on the temporal bones of patients with vestibular schwannoma reported the loss of inner and outer hair cells, cochlear neural loss, and precipitate in the perilymph and endolymph in the cochlea on the affected side [6]. The present study showed no relationship between vertigo and the signal intensity of the vestibular perilymph. We also did not clarify the relationship between vertigo and endolymphatic hydrops of the vestibule.

However, this does not necessarily conclude any correlation between dysfunctions in the vestibule and semicircular canals and endolymphatic abnormalities. Vestibular and semicircular canal function may be assessed using several different techniques including the caloric test, head impulse test (HIT), and vestibular evoked myogenic potentials (VEMP). Jerin et al. reported that a patient with vestibular schwannoma and vertigo attack exhibited vestibular hydrops on postcontrast MR imaging, and changes in frequency tuning by ocular VEMP suggestive of endolymphatic hydrops [29]. Another study revealed a relationship between saccular hydrops on the unaffected side and vertigo in patients with vestibular schwannoma using non-contrast FIESTA-C [30]. Further research is warranted to examine the relationship between dysfunctions in the vestibule and semicircular canals and endolymphatic abnormalities.

3D FLAIR provides high sensitivity to subtle enhancement caused by lower concentrations of gadolinium than T1W images [16]. 3D FLAIR with contrast material was recently used to discriminate between the perilymph and endolymph; gadolinium-based contrast material administered intratympanically or intravenously was mainly distributed in the perilymph, resulting in increased signal intensity, whereas the endolymph showed low signal intensity. As a result, endolymphatic hydrops may be evaluated in patients with various diseases including Ménière's disease. Furthermore, Naganawa et al. reported the utility of heavily T2W 3D FLAIR combined with 3 Tesla MR imaging and the 32-channel head coil to assess endolymphatic hydrops in patients with Ménière's disease after the single-dose intravenous administration of contrast material [31]. Heavily T2W 3D FLAIR has the potential to detect low concentrations of gadolinium-based contrast material more sensitively than conventional 3D FLAIR, resulting in the separate visualization of the endolymph and perilymph after the single-dose administration of contrast material in patients with Ménière's disease. In addition, these authors developed subtraction images with heavily T2W 3D IR images or MRC [17]. Heavily T2W 3D IR allows for positive endolymph signal by shortening the inversion time of heavily T2W 3D FLAIR. By subtracting heavily T2W 3D IR or MRC from heavily T2W 3D FLAIR to create HYDROPS or HYDROPS2 images, respectively, it became possible to improve the CNR between the endolymph and perilymph and to more easily recognize the endolymphatic space.

The present study had several limitations. This was a retrospective study with a small sample size. A prospective study with a large number of patients is needed to evaluate the inner ear of vestibular schwannoma on MR imaging. Furthermore, vestibular schwannomas were not confirmed by histological studies, except for one case. However, we consider that MR findings typical of vestibular schwannoma did not lead to serious limitations. In addition, we did not prove the diagnosis of endolymphatic hydrops histologically because histological specimens were not obtained from living tissues. We also did not establish the optimal inversion time to suppress the signal of the endolymph on PPI and that of the perilymph on PEI. Another limitation is that patients with vestibular schwannoma were not compared with normal control subjects. Additionally, although our study was cross-sectional, some cases showed that MR features of the perilymph and endolymph may chronologically change. Therefore, longitudinal studies are needed to elucidate the relationship between serial alterations in function and the MR features of the inner ear.

## Conclusions

We analyzed signal and morphological changes in the vestibular and semicircular canal endolymph of patients with vestibular schwannoma on non-contrast 3D FLAIR. This method is useful for the evaluation of the inner ear without gadolinium-based contrast agents, and will provide insights into the pathology and treatment of this disease. A correlation was not observed between vertigo and MR manifestations of the vestibule. Further research is warranted to clarify the correlation between endolymphatic changes and vertigo.

## Data Availability

The datasets generated and/or analyzed during the current study are not publicly available due to their containing information that could compromise patient privacy and confidentiality, but are available from the corresponding author on reasonable request.

## Abbreviations

CI: Confidence interval
CNR: Contrast noise ratio
CSF: Cerebrospinal fluid
FIESTA-C: Fast imaging employing steady state acquisition with cycle phase
HIT: Head impulse test
HYDROPS: Hybrid of reversed image of positive endolymph signal and native image of positive perilymph signal
ICC: Intraclass correlation coefficient
IR: Inversion recovery
MRC: MR cisternography
PEI: Positive endolymphatic images
PPI: Positive perilymphatic images
ROI: Region of interest
SD: Standard deviation
SIR: Signal intensity ratio
TR: Repetition time
T1W: T1-weighted
T2W: T2-weighted
VEMP: Vestibular evoked myogenic potentials
